# Comparative Study of AI Modes in Ultrasound Diagnosis of Breast Lesions

**DOI:** 10.3390/diagnostics15050560

**Published:** 2025-02-26

**Authors:** Yu-Ting Hong, Zi-Han Yu, Chen-Pin Chou

**Affiliations:** 1 Radiology Department, Kaohsiung Veterans General Hospital, Kaohsiung 813414, Taiwan; rainstop28@gmail.com (Y.-T.H.); wanjhenyu@gmail.com (Z.-H.Y.); 2 Department of Radiology, Jiannren Hospital, Kaohsiung 813414, Taiwan; 3 Department of Medical Laboratory Science and Biotechnology, Fooyin University, Kaohsiung 831301, Taiwan; 4 Department of Pharmacy, College of Pharmacy, Tajen University, Pingtung 907101, Taiwan

**Keywords:** S-detect, breast imaging reporting and data system (BI-RADS), breast ultrasound, artificial intelligence, breast cancer

## Abstract

**Objectives:** This study evaluated the diagnostic performance of the S-Detect ultrasound system’s three selectable AI modes—high-sensitivity (HSe), high-accuracy (HAc), and high-specificity (HSp)—for breast lesion diagnosis, comparing their performance in a clinical setting. **Methods:** This retrospective analysis evaluated 260 breast lesions from ultrasound images of 232 women (mean age: 50.2 years) using the S-Detect system. Each lesion was analyzed under the HSe, HAc, and HSp modes. The study employed ROC curve analysis to comprehensively compare the diagnostic performance of the AI modes against radiologist diagnoses. Subgroup analyses focused on the age (<45, 45–55, >55 years) and lesion size (<1 cm, 1–2 cm, >2 cm). **Results:** Among the 260 lesions, 73% were identified as benign and 27% as malignant. Radiologists achieved a sensitivity of 98.6%, specificity of 64.2%, and accuracy of 73.5%. The HSe mode exhibited the highest sensitivity at 95.7%. The HAc mode excelled with the highest accuracy (86.2%) and positive predictive value (71.3%), while the HSp mode had the highest specificity at 95.8%. In the age-based subgroup analyses, the HAc mode consistently showed the highest area under the curve (AUC) across all categories. The HSe mode achieved the highest AUC (0.726) for lesions smaller than 1 cm. In the case of lesions sized 1–2 cm and larger than 2 cm, the HAc mode showed the highest AUCs of 0.906 and 0.776, respectively. **Conclusions:** The S-Detect HSe mode matches radiologists’ performance. Alternative modes provide sensitivity and specificity adjustments. The patient age and lesion size influence the diagnostic performance across all S-Detect modes.

## 1. Introduction

Contemporary breast cancer diagnostics are rapidly evolving, integrating cutting-edge technologies such as artificial intelligence and liquid biopsy [1,2,3,4]. For example, recent developments in deep learning have enabled the creation of AI algorithms that significantly enhance diagnostic performance [2,3,4]. While computer-aided detection (CADe) in mammography primarily focuses on identifying suspicious areas, breast ultrasound computer-aided diagnosis (CADx) systems are designed to differentiate benign and malignant masses [5,6]. Advancements in this technology enable early-stage disease diagnosis and treatment, significantly improving therapeutic outcomes [7,8].

A previous study evaluating the impact of Koios DS CADx software (v1.3, Koios Medical, Inc., Chicago, IL, USA), including the original, high-sensitivity, and high-specificity modes, on breast ultrasound interpretation demonstrated that while the original CADx interpretation did not significantly influence the diagnostic performance, the high-specificity mode enhanced radiologists’ accuracy and efficiency [9]. S-Detect also provides three selectable modes: high-sensitivity (HSe), high-accuracy (HAc), and high-specificity (HSp). The HAc mode, which balances sensitivity and specificity, is the most commonly used mode in S-detect research [10,11]. As an AI-assisted ultrasound system, S-Detect with the HAc mode has demonstrated promising efficacy in breast cancer detection [10,11].

A meta-analysis showed that S-Detect achieved 82% sensitivity and 83% specificity in differentiating benign and malignant breast masses [7]. However, outcomes may vary according to patient characteristics and geographic factors; for example, one study observed higher S-Detect sensitivity among North American and Korean radiologists compared with their European counterparts [12].

The accuracy of AI tools in breast cancer diagnosis could be affected by the tumor size and breast density [13]. The sensitivity of ultrasound for detecting breast cancer is higher in older women (78.7%) than in younger women (69.2%), but the specificity is lower (90.2% vs. 82.9%) [8]. The use of computer-aided diagnosis (CAD) is an effective method for breast tumor detection, especially for lesions ≤2 cm in size [14,15,16]. Whether S-Detect should be selectively applied or a single AI diagnostic approach should be used across all lesions remains uncertain [15]. This study evaluated three AI modes for breast cancer detection, examining how the patient age and tumor size influence diagnostic performance.

## 2. Materials and Methods

### 2.1. Patient Selection

This retrospective study, conducted with approval from the Institutional Review Board of Kaohsiung Veterans General Hospital (IRB number: KSVGH23-CT2-03), enrolled 354 women who underwent breast ultrasounds between October 2018 and May 2022. The IRB waived informed consent because this study analyzed previously collected data without patient identifiers. The inclusion criteria comprised the following: (1) women aged 20 years or older, (2) individuals presenting with breast-related symptoms such as palpable lumps, pain, or nipple discharge, and (3) those who self-referred for a screening ultrasound. Previously collected ultrasound scans of suspicious breast masses were re-evaluated by an AI system. A total of 122 cases were excluded due to missing data: 41 cases lacked essential images required for AI analysis, 20 had incomplete clinical histories, and 61 did not have adequate 24-month imaging follow-up data, which were critical for diagnostic validation. Consequently, 232 patients with a total of 260 lesions were eligible for further assessment.

### 2.2. Ultrasound Procedure

In this study, breast ultrasounds were performed using the RS85 system (Samsung Medison Co., Ltd., Seoul, Republic of Korea), which has a 3–12 MHz linear array transducer (L3-12A). The ultrasounds were performed by board-certified sonographers with 10–25 years of experience. When a mass lesion of interest was found, images showing the longest diameter of the lesion were captured and stored for later analysis. The archived ultrasound images were interpreted by one of six experienced breast radiologists, each with 5–26 years of experience interpreting breast ultrasounds. The radiologists used the 5th edition of the American College of Radiology (ACR) guidelines to categorize the lesions into BI-RADS categories 1–5.

### 2.3. Imaging Selection

**Figure 1:**
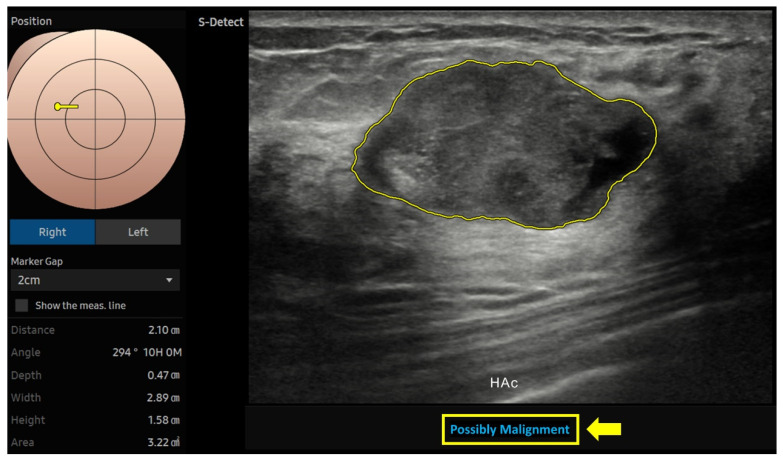
An AI-generated region of interest (ROI) was delineated around the mass’s border. S-Detect automatically extracted and displayed ultrasound features based on the ultrasound BI-RADS lexicon. The final determination based on the high-accuracy (HAc) mode was categorized as either “possibly benign” or “possibly malignant” (arrow).

Using still images for data collection from S-Detect, a single radiologist, who had previously analyzed the ultrasound, reapplied S-Detect to the same image used in the B-mode grayscale ultrasound for each breast mass. A region of interest (ROI) was delineated around the mass’s border, auto-generated by the AI system after manual selection, as S-Detect requires operator-defined input and does not independently detect lesions [17,18]. S-Detect automatically generated ultrasound features and final diagnostic classifications based on the BI-RADS lexicon. These outputs were recorded for subsequent data analysis. Each breast mass was analyzed using all three AI modes of S-Detect, with final classifications assigned in a dichotomous manner as either ‘possibly benign’ (negative) or ‘possibly malignant’ (positive), based on the AI-generated assessment (Figure 1) [17,18].

### 2.4. AI Analysis

S-Detect enhances diagnostic performance using deep learning and an advanced Fully Convolutional Network and lesion-centered preprocessing for precise segmentation. For benign versus malignant classification, a modified GoogLeNet architecture is employed, designed to process grayscale ultrasound images and generate a binary classification output [17]. The classification system comprises three networks, each optimized for distinct sensitivity thresholds. The softmax outputs from these networks are subsequently processed using logical operations: an “OR” operation enhances sensitivity in the HSe mode by reducing false negatives, “Direct Classification” maintains balanced diagnostic accuracy in the HAc mode, and an “AND” operation in the HSp mode increases specificity by minimizing false positives [17].

In the study, the diagnostic performance of Samsung’s ultrasound S-Detect software (V2) was thoroughly evaluated. The software was used to assess the enrolled breast lesions using three vendor-provided AI algorithmic modes. Each of these modes is designed for different diagnostic objectives and has unique implications for the overall accuracy of the assessment. Specifically, the HSe mode prioritizes the identification of true positive cases, meaning that it excels at detecting the majority of individuals who have the disease. Conversely, the HSp mode places an emphasis on specificity, meaning that it minimizes false positives and effectively identifies individuals who do not have the disease. The HAc mode aims for a balanced approach, focusing on both sensitivity and specificity, to provide an accurate diagnosis for both affected and unaffected individuals.

**Figure 2:**
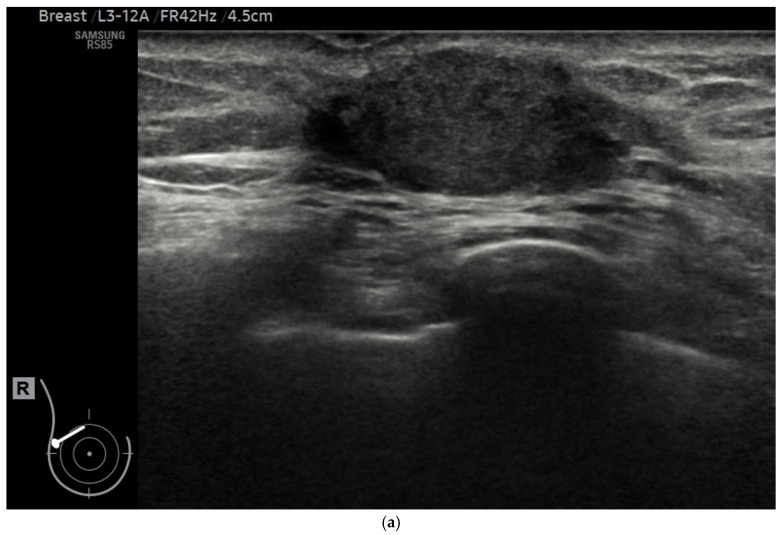

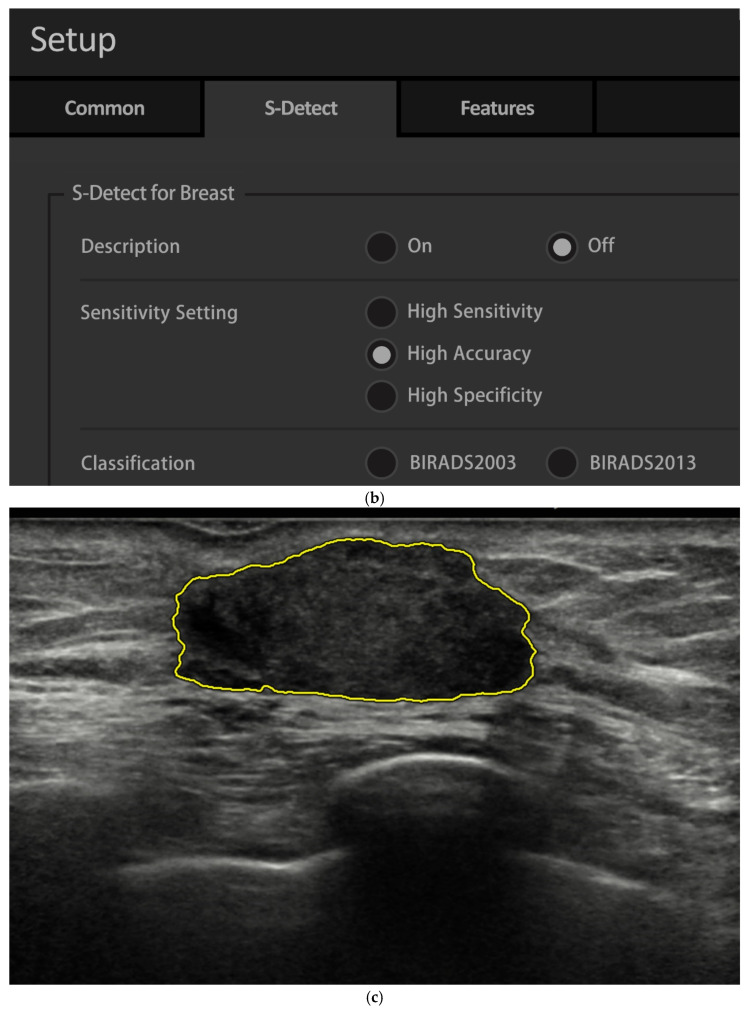
A 61-year-old woman diagnosed with invasive breast cancer. (**a**) A greyscale breast ultrasound serving as an example of how AI software (V2) can evaluate a suspicious lesion through three AI modes. (**b**) The S-detect system on the ultrasound machine provides three distinct AI modes: high-sensitivity (HSe), high-accuracy (HAc), and high-specificity (HSp). (**c**) Utilizing AI capabilities, the lesion of interest is automatically outlined when a sonographer places a marker within the targeted area.

For each lesion enrolled in the study, assessments were conducted in all three aforementioned AI modes. The software generated evaluations categorizing each lesion as either benign or malignant, and these results were carefully recorded for further analysis (Figure 2a–c).

### 2.5. Diagnosis

For the clinical study, biopsies and subsequent histopathological evaluations were obtained for 160 breast mass lesions. An additional 100 lesions were classified as ’probably benign’ and were managed with a short-term follow-up without a biopsy. After 24-month ultrasound surveillance, these “probably benign” lesions showed no significant changes, confirming their initial clinical diagnosis as benign. The diagnostic performance of the S-Detect system was assessed against the final clinical diagnoses, which were based on either the histopathological findings from the biopsies or the absence of any detectable abnormalities at the 24-month follow-up.

### 2.6. Performance Metrics

This study primarily emphasized the evaluation of disease classification accuracy, complemented by a comprehensive analysis of additional diagnostic metrics, including the sensitivity, specificity, and precision [19]. This multifaceted approach highlights the complexity of evaluating machine learning algorithms in medical settings [17]. Contemporary medical technology varies in the evaluation metrics used for machine learning algorithms. The F1 score is particularly valuable in evaluating machine learning algorithms, as it harmonizes precision and recall (sensitivity) to provide a single, balanced measure of performance [17,19]. An F1 score closer to 1 indicates a superior diagnostic ability, effectively capturing both the proportion of correctly identified positive cases and the model’s ability to minimize false positives [17,20]. In this study, we conducted external validation using real-world clinical data obtained from a single institution. Unlike internal cross-validation, which is primarily used for model optimization in controlled settings, external validation evaluates the model’s performance on an independent dataset that reflects real-world clinical scenarios [21].

### 2.7. Statistical Analysis

Statistical analyses were conducted using SPSS 22.0 and MedCalc software (Version 22.009) to calculate diagnostic metrics and compare outcomes. Diagnostic metrics, including the sensitivity, specificity, accuracy, positive predictive value (PPV), negative predictive value (NPV), and F1 score were calculated for comparisons. The diagnostic performance was assessed by creating an ROC curve and calculating the AUC values, with the significance set at *p* < 0.05.

## 3. Results

### 3.1. Patient Demographics and Clinical Data

Table 1 summarizes the clinical demographics of the 232 patients. Among them, 53% were aged ≤50 years, and 59% were premenopausal. The breast involvement was bilateral in 6% of cases, while 90% had a single lesion and 10% had multiple lesions. The primary indications for breast ultrasound were clinical symptoms (71%) and self-referred screening (29%).

### 3.2. Breast Lesion Imaging Features

Table 2 details the imaging characteristics of the 260 breast lesions, 73% of which were benign and 27% malignant. A biopsy was performed in 61.5% of cases. Most lesions were oval-shaped (57.7%) with circumscribed margins (50%). The lesion sizes were distributed as follows: >2 cm (26%), 1–2 cm (44%), and ≤1 cm (30%). The most common BI-RADS classification was Category 4, which indicates a variable risk of malignancy but also a high frequency of benign outcomes.

### 3.3. Biopsy Histopathologic Findings

Table 3 provides details on the pathology of 160 biopsied breast lesions. Among the 90 benign lesions, 44% were fibroadenomas, 36% exhibited fibrocystic changes with usual ductal hyperplasia, and the remaining 20% encompassed other benign conditions. Of the 70 malignant lesions, 81% were invasive ductal carcinoma (IDC), with the remainder comprising ductal carcinoma in situ (DCIS) and invasive lobular carcinoma (ILC). Overall, the study evaluated 260 lesions, including 100 with a two-year follow-up confirming benignity and 160 that underwent biopsy, of which 70 (44%) were malignant.

### 3.4. Radiologist vs. AI Performance

**Figure 3:**
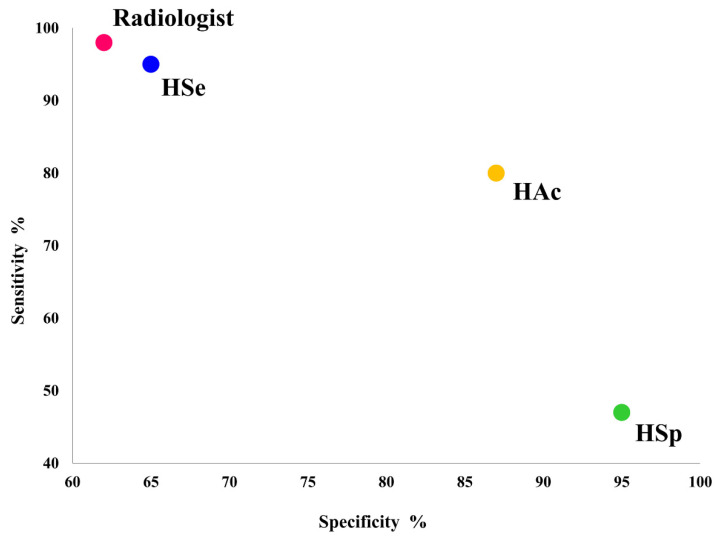
Radiologists and three AI modes (high-sensitivity (HSe), high-accuracy (HAc), and high-specificity (HSp)) had varying diagnostic performance in terms of sensitivity and specificity for breast ultrasound mass lesions. The HSe mode closely aligned with radiologists’ performance.

The radiologists achieved a diagnostic performance benchmark with a sensitivity of 98.6%, specificity of 64.2%, accuracy of 73.5%, PPV of 50.7%, NPV of 99.2%, AUC of 0.813, and F1 score of 0.668. The diagnostic efficacy of three artificial intelligence (AI) modes—HSe, HAc, and HSp—was assessed in our study using a sample of 260 breast lesions. The diagnostic accuracy of the AI modes and radiologists in breast ultrasound lesion diagnosis varied considerably, with the AI modes generally exhibiting superior specificity but inferior sensitivity (Figure 3). For example, the HSe mode classified 51% of the lesions as benign and 49% as malignant. In contrast, benign lesion classifications were more pronounced in the HAc and HSp modes, at 69% and 85%, respectively.

As shown in Table 4, the HSe mode had the highest sensitivity at 95.7% (95% CI: 87.9–99.1). The HAc mode had the highest accuracy, at 86.2%, while the HSp mode had the highest specificity at 95.8%. When examining the sensitivity spectrum of all the AI modes, the values ranged from 45.7% to 95.7%. While these were generally lower than the radiologists’ sensitivity, the statistical significance varied, with *p*-values ranging from 0.049 to <0.001. Importantly, both the HSp and HAc modes significantly outperformed the radiologists in terms of specificity, as indicated by all *p*-values being less than 0.001.

Among the AI algorithms evaluated for breast lesion analysis, the HAc mode was the most accurate, followed by the HSe mode, the radiologists, and lastly the HSp mode. However, the HSp mode, while leading in specificity, had the lowest sensitivity.

### 3.5. F1 Score: Radiologists vs. AI

The radiologists achieved a sensitivity of 98.6% and a specificity of 64.2%, yielding an F1 score of 0.668 (Table 4). This indicates that while radiologists excelled in identifying malignant lesions, their lower specificity contributed to a higher rate of false positives. The HSe mode demonstrated a marginally higher F1 score of 0.678, with a sensitivity of 95.7% and a specificity of 68.4%, indicating strong performance in detecting malignant lesions with a slightly reduced rate of false positives compared to the radiologists. The HAc mode excelled with the highest F1 score of 0.760, showing a balanced sensitivity of 81.4% and specificity of 87.9%. This means that the HAc mode was able to correctly identify both malignant and benign lesions with a high degree of accuracy. The HSp mode scored the lowest F1 score of 0.579 due to its emphasis on specificity (95.8%) at the expense of sensitivity (45.7%). This means that the HSp mode was very good at identifying benign lesions, but it had a high rate of false negatives.

### 3.6. AUC: Radiologist vs. AI Accuracy

**Figure 4:**
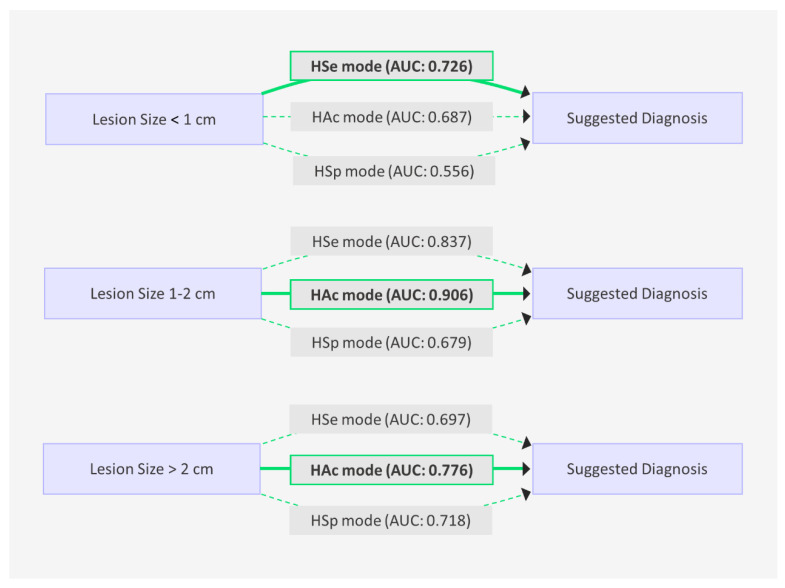
Diagnostic performance of the three AI modes in breast lesion analysis stratified by the lesion size (<1 cm, 1–2 cm, and >2 cm). Performance was assessed using the area under the curve (AUC).

**Figure 5:**
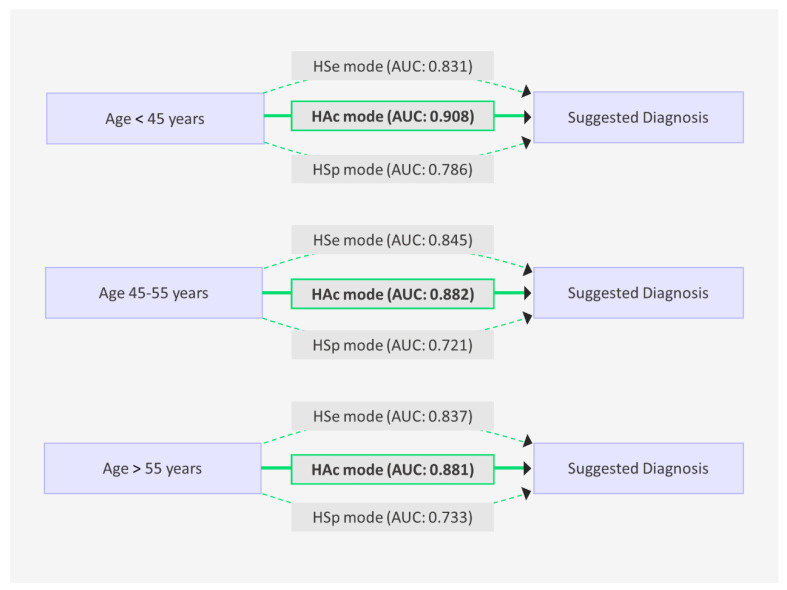
Diagnostic performance of the three AI modes in breast lesion analysis stratified by patient age groups (<45 years, 45–55 years, and >55 years). Performance was assessed using the area under the curve (AUC).

Figure 4 and Figure 5 provide a comprehensive comparison of the diagnostic performance of the AI modes and experienced radiologists. The HAc mode demonstrated the highest diagnostic accuracy, achieving an AUC of 0.841 (95% CI: 0.79–0.88), followed by the HSe mode and the radiologist group, each with an AUC of 0.813. The HSp mode recorded the lowest AUC at 0.704 (95% CI: 0.65–0.76). These results indicate that the HAc mode was the most effective in classifying breast lesions as benign or malignant, outperforming the HSe mode, the radiologists, and the HSp mode in the overall diagnostic accuracy.

### 3.7. Performance by Age and Size

The HAc mode consistently outperformed the other modes across all age categories. For women under 45, the HAc mode achieved an AUC of 0.908 (95% CI: 0.83, 0.96), whereas the HSp mode lagged behind with an AUC of 0.786. In women aged 45–55, the HAc mode continued its superior performance, registering an AUC of 0.882 (95% CI: 0.79, 0.95), compared to the HSp mode’s AUC of 0.721. For women aged 55 and above, the HAc mode recorded an AUC of 0.881, while the HSp mode had an AUC of 0.733.

Considering the lesion size, the HSe mode exhibited the highest AUC (0.726) for lesions smaller than 1 cm. For lesions ranging from 1 to 2 cm, the HAc mode achieved an AUC of 0.906, and for those larger than 2 cm, it recorded an AUC of 0.776, maintaining its superior diagnostic performance across lesion sizes.

## 4. Discussion

The Samsung S-Detect ultrasound AI system offers three diagnostic modes—HSe, HAc, and HSp—designed for distinct diagnostic objectives. Our findings confirm that these modes align with their manufacturer-defined purposes. The HSe mode demonstrated a sensitivity of 95.7%, consistent with the >95% sensitivity criterion, while the HSp mode achieved a specificity of 95.8%, meeting its >95% specificity benchmark. The HAc mode provided a balanced approach, excelling in both sensitivity and specificity for lesion classification. For lesions smaller than 1 cm, the HSe mode demonstrated the highest diagnostic efficacy, with an AUC of 0.726. In contrast, for lesions larger than 1 cm and across all age groups, the HAc mode consistently outperformed other modes, achieving an AUC of up to 0.908. These findings validate the manufacturer’s specifications and provide evidence-based guidance for tailoring AI mode selection to patient-specific factors, such as the lesion size and age.

As a comparison with mammography-based AI [22,23], this study highlights differences in analysis algorithms, AI parameters, and diagnostic trade-offs for ultrasound-based breast cancer detection. Notably, a class imbalance in the training/validation/test split and external validation dataset may have impacted the model performance, particularly for malignant lesions [22,23]. This imbalance likely contributed to reduced sensitivity and F1 scores for malignant lesion classification. Future work should include diverse use case studies to ensure generalizability across clinical settings.

Choi et al. have reported performance metrics for the HAc mode, including 85% sensitivity, 95.4% specificity, and 92.1% overall diagnostic accuracy [17]. These findings highlight the HAc mode’s reliability for clinical application and align closely with its performance observed in our study. This suggests that the HAc mode can be used in most clinical practices [17]. In our study, the HAc mode was found to have lower sensitivity but higher accuracy and specificity than radiologists. This is consistent with the findings of previous studies [17] and suggests that the S-Detect default HAc mode setting can achieve a level of agreement comparable to that of radiologists.

The S-Detect system utilized 7408 ultrasound breast images from 5151 patients, comprising 4254 benign lesions (57.4%; e.g., cysts, fibroadenomas) and 3154 malignant lesions (42.6%; e.g., carcinomas), to train a GoogLeNet-based CNN for binary classification [17]. By leveraging a large, biopsy-proven dataset and a standard CNN architecture, a study found that it demonstrated strong performance, with an AUC > 0.9, and a robust discriminative ability [17]. Our study provides real-world external validation of an AI-driven ultrasound diagnostic system, focusing on key deep learning performance metrics.

S-Detect outperformed radiologists in terms of the specificity, positive predictive value, and overall accuracy in breast ultrasound evaluations [24]. The AI use case also achieved a higher area under the ROC curve, suggesting its potential as an adjunct diagnostic tool to improve ultrasound specificity for breast tumors [24,25]. Additionally, S-Detect significantly enhanced the inter-reader consistency in lesion assessment according to the BI-RADS US lexicon, with a *p*-value of <0.0001 [18]. Both Kim et al.’s study and our findings highlight AI’s potential to outperform radiologists in specificity and accuracy, although with variations in sensitivity, thereby underscoring the potential of integrating AI modes like HSe, HAc, and HSp as complementary tools in breast imaging diagnostics.

A study found that an AI program for breast cancer diagnosis was in concordance with the readings of 10 radiologists across 299 breast ultrasound images with suspicious lesions [12]. The AI program’s sensitivity, specificity, and accuracy were above 0.8, in agreement with the highest performing radiologists, indicating its potential to improve the ultrasound breast cancer diagnosis accuracy of most radiologists [12]. In another study involving 100 breast ultrasound images from 91 women, they compared the diagnostic performance between experienced and less experienced radiologists [26]. Less experienced radiologists had a significantly improved NPV with CAD assistance. Experienced radiologists had significantly improved specificity and PPVs with CAD assistance [26]. Our results indicate that the HAc mode, with its high PPV and specificity, is particularly beneficial for experienced radiologists aiming to optimize diagnostic precision. Conversely, the HSe mode, with its superior NPV, may better support less experienced radiologists by reducing the likelihood of false negatives, thereby enhancing diagnostic confidence [27].

In the evaluation of small breast lesions, the S-Detect default mode demonstrated superior performance for lesions with sizes smaller than 2 cm [14,28]. S-Detect correctly diagnosed 15.9% more small malignant lesions than an experienced ultrasound radiologist [28]. Furthermore, the greatest diagnostic performance for evaluating small breast lesions was achieved with a combination method incorporating radiologists and S-Detect, resulting in an increase of 19.9% in the correct detection of small cancers [28]. Another study of small breast lesions found that less experienced radiologists had a significant improvement in diagnostic performance when using S-Detect, with the AUC increasing from 0.74 to 0.88. Experienced radiologists also benefited from using S-Detect, but the improvement was smaller (the AUC increased from 0.84 to 0.90). The benefit of S-Detect diminished for lesions larger than 2 cm, where the AUC slightly decreased for both groups [14]. Our study showed lower AUC values for lesions larger than 2 cm, regardless of the mode (HSe, HAc, or HSp) or the radiologists. This suggests that the diagnostic performance of AI systems for breast lesions larger than 2 cm is diminished.

Breast cancer that is less than 1 cm in size is clinically significant because it is often an early-stage cancer with a lower risk of spreading and a better prognosis [29,30]. Our study found that the HSe mode had better performance than the other two modes for lesions less than 1 cm in size, and the radiologist had the best performance. Conversely, for larger tumors, the HAc mode, which balances sensitivity and specificity, is the better choice.

A previous study by Devolli-Disha et al. found that ultrasound imaging is more sensitive in detecting breast cancer in women under 45 years old with dense breast tissue [8]. Our study found that ultrasound imaging was more sensitive in women under 45 years old, with the HAc mode having the highest AUC of 0.908. However, the AUC decreased in women between the ages of 45 and 55 for most modes and radiologists, suggesting that breast cancer is more difficult to diagnose in this group. This could be due to changes in the breast tissue composition, hormonal fluctuations, and other physiological factors [8].

Our research found that the HAc mode generally performed the best, making it suitable for a variety of clinical applications. However, the HSe mode may be more appropriate in situations where missing a diagnosis is unacceptable. The F1 score, a metric that balances sensitivity and specificity, can be particularly helpful in these cases. Currently, systems only allow one AI mode to be available at a time, which limits clinical flexibility. This is a disadvantage since practitioners should ideally have easy access to a suitable mode for making accurate decisions [31].

This study has several limitations, including its retrospective design, variability in the expertise of participating radiologists and sonographers, and the lack of histopathological confirmation for some benign lesions. These factors may have influenced the findings and limited the generalizability of the results. The lower F1 score observed in this study may be attributed to several real-world diagnostic challenges. For instance, 30% of the lesions were smaller than 1 cm, making them harder to classify accurately. Additionally, 48% of the lesions had suspicious margins, and some benign cases mimic malignancy, particularly in the study’s age range, further complicating the diagnostic process. Since S-Detect requires manual lesion selection and does not independently detect abnormalities, control images without lesions could not be evaluated, limiting the assessment of false-positive detections [2,32]. The study was primarily conducted in a medical center and a veterans’ hospital, with participants having a mean age of 50.2 years, which could have influenced the outcomes. However, the radiologists and sonographers were well experienced, and all benign lesions without a biopsy underwent a 24-month imaging follow-up. Future studies should adopt a prospective design to reduce bias, standardize the assessment protocols across participants, and ensure histopathological confirmation for all benign lesions. Expanding evaluations to include multiple ultrasound system manufacturers and collaborating with AI developers could further enhance the robustness of findings. Additionally, establishing a large, diverse, case-specific database would provide more generalizable insights into AI performance in breast cancer diagnostics [33].

## 5. Conclusions

The S-Detect HSe mode matches radiologists’ diagnostic performance in breast ultrasound analysis, while the HAc mode improves accuracy and the HSp mode exhibits enhanced specificity. The diagnostic efficacy across all modalities is affected by the patient age and lesion size.

## Figures and Tables

**Table 1:** Demographic data of patients (n = 232).

Characteristic	n (%)
**Total**	**232**
**Age, y**	
Mean ± SD	50.2 ± 14.2
Median, range	48, 19–87
>50	108 (47)
≤50	124 (53)
**Menopausal status**	
Premenopausal state	137 (59)
Postmenopausal state	95 (41)
**Laterality of lesion**	
Left breast	116 (50)
Right breast	103 (44)
Bilateral breasts	13 (6)
**Lesion number per patient**	
1	209 (90)
2	18 (8)
≥3	5 (2)
**Purpose of exam**	
Screening	67 (29)
Diagnosis	165 (71)
**Methods of diagnosis**	
Biopsy	147 (63)
Follow-up (24 months)	85 (37)

Note: Data in parentheses represent percentages.

**Table 2:** Clinical information on lesions (n = 260).

Characteristic	n (%)
**Final diagnosis**	
Malignant	70 (27)
Benign	190 (73)
**Diagnosis method**	
Biopsy	160 (62)
24-month follow-up	100 (38)
**Shape**	
Oval	150 (58)
Round	21 (8)
Irregular	89 (34)
**Margin**	
Circumscribed	128 (50)
Indistinct	49 (19)
Angular	7 (2)
Microlobulated	57 (22)
Spiculated	19 (7)
**Lesion size (cm)**	
<1	79 (30)
≥1, ≤2	113 (44)
>2	68 (26)
**BI-RADS determined by radiologists**	
2	59 (23)
3	63 (24)
4	133 (51)
5	5 (2)

Note: Data in parentheses represent percentages.

**Table 3:** Histopathology of biopsied breast lesions (n = 160).

Characteristics	n (%)
**Benign (n = 90)**	
Fibroadenoma	40 (44)
FCD (fibrocystic changes)	32 (36)
Inflammatory breast disease	5 (6)
Papilloma	4 (4)
Phyllode tumor	2 (2)
Other benign pathology	7 (8)
**Malignant (n = 70)**	
IDC (invasive ductal carcinoma)	57 (81)
ILC (invasive lobular carcinoma)	5 (7)
DCIS (ductal carcinoma in situ)	2 (3)
Papillary ductal carcinoma in situ	2 (3)
Other invasive cancer	4 (6)

Note: Data in parentheses represent percentages.

**Table 4:** Comparative analysis of diagnostic performance between radiologists and three AI modes: HSe, HSp, and HAc (n = 260).

Mode	Sensitivity	Specificity	Accuracy	PPV	NPV	F1 Score
Rad	98.6 (92.4–100)	64.2 (56.7–70.8)	73.5 (67.7–78.7)	50.7 (45.9–55.5)	99.2 (94.5–100)	0.668 (0.588–0.737)
HSe	95.7 (87.9–99.1) *p* = 0.049 *	68.4 (61.3–75) *p* = 0.288	75.8 (70.1–80.9) *p* = 0.545	52.8 (47.4–58.1) *p* = 0.643	97.7 (95.6–99.8) *p* = 0.183	0.678 (0.599–0.751)
HAc	81.4 (70.3–89.7) *p* < 0.001 ***	87.9 (82.4–92.2) *p* < 0.001 ***	86.2 (81.4–90.1) *p* < 0.001 ***	71.3 (62.4–78.7) *p* < 0.001 ***	92.8 (88.7–95.5) *p* < 0.001 ***	0.760 (0.677–0.832)
HSp	45.7 (33.7–58.1) *p* < 0.001 ***	95.8 (91.9–98.2) *p* < 0.001 ***	82.3 (77.1–86.8) *p* = 0.015 **	80.0 (66.0–89.2) *p* < 0.001 ***	82.7 (79.4–85.6) *p* < 0.001 ***	0.579 (0.465–0.685)

Note: HSe: High-Sensitivity Mode; HAc: High-Accuracy Mode; HSp: High-Specificity Mode; NPV: Negative Predictive Value; PPV: Positive Value; Rad: Radiologists. Data in parentheses represent the range of the 95% confidence interval. Statistical significance: * *p* < 0.05, ** *p* < 0.01, *** *p* < 0.001. *p*-values are in comparison to the Rad.

## Data Availability

The data that support the findings of this study are available from Kaohsiung Veterans General Hospital IRB, but restrictions apply to the availability of these data, which were used under license for the current study and so are not publicly available. Data are, however, available from the authors upon reasonable request and with the permission of Kaohsiung Veterans General Hospital IRB.

## Abbreviations

AI (artificial intelligence), HSe (high-sensitivity), HAc (high-accuracy), HSp (high-specificity), and AUC (area under the ROC curve).
